# Modeling of Material Removal Rate for the Fixed-Abrasive Double-Sided Planetary Grinding of a Sapphire Substrate

**DOI:** 10.3390/ma17153688

**Published:** 2024-07-25

**Authors:** Gen Chen, Zhongwei Hu, Lijuan Wang, Yue Chen

**Affiliations:** 1 Institute of Manufacturing Engineering, Huaqiao University, Xiamen 361021, China; 22013080001@stu.hqu.edu.cn (G.C.); wljxmhy@163.com (L.W.); hquchenyue@163.com (Y.C.); 2 Institute of Mechanical Engineering and Automation, Huaqiao University, Xiamen 361021, China; 3 Intelligent Manufacturing College, Xiamen City University, Xiamen 361021, China

**Keywords:** removal rate model, trajectory length, double-sided planetary grinding, sapphire

## Abstract

Double-sided planetary grinding (DSPG) with a fixed abrasive is widely used in sapphire substrate processing. Compared with conventional free abrasive grinding, it has the advantages of high precision, high efficiency, and environmental protection. In this study, we propose a material removal rate (*MRR*) model specific to the fixed-abrasive DSPG process for sapphire substrates, grounded in the trajectory length of abrasive particles. In this paper, the material removal rate model is obtained after focusing on the theoretical analysis of the effective number of abrasive grains, the indentation depth of a single abrasive grain, the length of the abrasive grain trajectory, and the groove repetition rate. To validate this model, experiments were conducted on sapphire substrates using a DSPG machine. Theoretical predictions of the material removal rate were then juxtaposed with experimental outcomes across varying grinding pressures and rotational speeds. The trends between theoretical and experimental values showed remarkable consistency, with deviations ranging between 0.2% and 39.2%, thereby substantiating the model’s validity. Moreover, leveraging the insights from this model, we optimized the disparity in the material removal rate between two surfaces of the substrate, thereby enhancing the uniformity of the machining process across both surfaces.

## 1. Introduction

In recent years, under severe energy consumption and heavy environmental pressures, LEDs (light emitting diodes), as a true green light source, have been developed rapidly [1,2]. The core of LEDs lies in the LED chip, and the substrate is the basis for preparing high-end LED chips [3,4]. Substrate machining quality directly affects the quality and life of LED chips. More than 90% of LED substrates are made of sapphire, which must meet the requirements of ultra-flat, ultra-smooth, and non-damaged surfaces [5]. However, the characteristics of sapphire, such as high hardness, high brittleness, and remarkable anisotropy, results in great difficulties in its processing and manufacturing [6,7]. The mainstream production process of LED substrates mainly includes wire cutting, grinding, polishing, and other processes [8]. The primary goal of the grinding process is to eliminate surface damage from wire-cutting, enhance surface accuracy, reduce roughness, and prepare the workpiece for subsequent polishing [9,10,11]. In contrast with traditional free-abrasive, double-sided grinding, fixed-abrasive DSPG enables the rapid removal of surface material from sapphire substrates and flattening of the surface [12]. This method achieves a high material removal rate while maintaining processing quality [13].

Enhancing the *MRR* during the grinding stage of sapphire substrate processing is crucial for reducing the overall processing time. Consequently, predicting the *MRR* in the grinding process has become a significant area of interest for researchers. During grinding, the removal of material is influenced by various factors, such as processing equipment, process parameters, and external conditions [14]. Many scholars have attempted to establish a direct link between the grinding process parameters and material removal and to reveal the material removal mechanism, to some extent, through material removal models [15,16]. One of the more classical models is the empirical equation for the *MRR* summarized by Preston [17] when grinding with free abrasives, namely the Preston equation: MRR=K·P·V, where *MRR* is the rate at which material is removed from the surface of the workpiece, *K* is the Preston constant coefficient, *P* is the grinding pressure exerted on the surface of the workpiece by the grinding disc, and *V* is the rotation speed of the workpiece relative to the grinding disc. The Princeton equation shows that for free abrasive grinding, the *MRR* is linearly related to the grinding pressure applied by the wheel and the relative speed of the workpiece to the wheel, and the effects of other processing conditions on the *MRR* are included in the Princeton constant. Although widely used for modeling the *MRR* in the free-abrasive grinding of various materials, the Preston equation has limitations. Its accuracy in predicting the *MRR* is affected by the random motion of abrasive particles and processing conditions such as the grinding fluid flow rate.

To enhance the accuracy of *MRR* model predictions, numerous scholars have refined the Preston equation. They have incorporated influences of mechanical properties of abrasive grains, grinding disks, and workpieces, along with abrasive particle size, concentration, and loss. These modifications have been applied to the grinding processes of silicon wafers and sapphire, aiming for more precise outcomes. Liu et al. [18] took into account the physical properties of abrasive particles, grinding disks, and workpieces. The *MRR* equation in the silicon-wafer-grinding process was obtained based on the Preston equation. Tseng et al. [19] analyzed how the loss of abrasive particles on the tool’s surface and the excision marks on the workpiece affect the relative speed during the grinding process. They developed a model for the *MRR* in silicon wafer grinding using Preston’s equation. Zhou et al. [20] advanced the ultra-precision machining of silicon wafers by modifying the Preston equation. They introduced the effect of abrasive fluid’s relative flow on grinding pressure, enhancing the model’s accuracy and application. Lin et al. [21] utilized regression analysis to discuss the process parameters in the grinding process of sapphire substrates, and they incorporated the combined effects of abrasive particle size and concentration into the equation in order to enhance the predictive precision of the model.

The trajectory of abrasive particles is a key factor in material removal. However, most current research on *MRR* models focuses on free-abrasive grinding, influenced by the randomness of abrasive distribution and other variables. This research often overlooks the impact of abrasive particle distribution and motion patterns on material removal [22], highlighting the need to improve the accuracy of theoretical *MRR* models. When solidified abrasives are used for grinding, removal effects such as chemical interaction between the grinding fluid and the surface of the workpiece being processed can be ignored. Therefore, the material removal process by abrasive grains is considered a purely mechanical action [23]. Wang [24] proposed using semi-solidified diamond abrasives for sapphire grinding and derived the model of the *MRR* based on the abrasive and workpiece sizes, considering the receding of the semi-solidified abrasives and the contact model between the abrasive and the workpiece. The model considers that the parameters affecting the material removal rate during solidified abrasive processing include the grinding wheel speed, the concentration of abrasive contained in a unit volume of a grinding tool, the grain size of the abrasive, and the depth of penetration of the abrasives. Li et al. [25] considered the heat generation of the abrasive during the grinding process in double-sided grinding by using a single abrasive grain as a heat source and proposed a new material removal model with thermal-mechanical coupling based on the analysis of the abrasive grain motion trajectory. This model revealed that an uneven temperature distribution within the workpiece during double-sided grinding causes material removal inhomogeneity. Specifically, the temperature increases radially, causing significant axial deformation in high-temperature regions, which in turn causes a significant amount of material to be removed. Liu et al. [26] investigated the effect of process parameters (e.g., grinding duration, grit size of abrasive, abrasive concentration, grinding pressure, and grinding speed) on the *MRR* in sapphire wafer grinding. They further designed experiments and finally combined the results of experimental data with theoretical analysis to develop a regression model for predicting the *MRR*. Additionally, Lin et al. [27] explored a novel solidified abrasive grinding (NFAL) tool, using mathematical statistics, contact mechanics, kinematic theory, wear mechanism, and linear cumulative removal principles. They developed an *MRR* model and experimentally validated that the theoretical model accurately predicted the linear increase in the *MRR* with rising rotational speed and the increase in the *MRR* with higher applied loads. Zhao et al. [28] performed a theoretical analysis of the *MRR* for nanocomposite ceramics under two-dimensional ultrasonic vibration conditions. They established a mathematical model for the *MRR* during ultrasonic grinding and conducted quantitative calculations using MATLAB software. Their analysis demonstrated that, under specific conditions, the theoretical *MRR* of ultrasonic grinding exceeds that of conventional grinding by more than five times.

The review of the papers reveals that there are two main types of models of the *MRR*, one is the experience formula and the other is the model established based on theoretical analysis. The experience formula represented by Princeton’s formula presents difficulty in calculating the *MRR* of the upper and lower surfaces of the same substrate, and the model of the *MRR* established via theoretical analysis is not sufficient for the discussion of the problem of concern in this paper. Therefore, we propose the method of establishing a model of the *MRR* for the DSPG of sapphire substrates based on the trajectory equations of the abrasive grains and combined with the analysis of the contact mechanics between the abrasive grains and the substrate. In the fixed-abrasive DSPG, the distribution density and relative positioning of abrasive particles are predetermined since they are fixed on the grinding wheel. Consequently, the length of the grains’ trajectory with respect to the surface of the workpiece and the depth of cut of the abrasive ultimately determine the amount of material removed during the grinding process of fixed abrasive grains. Furthermore, in this paper, based on the relative motion trajectory length of grains relative to the sapphire substrate, the distribution of grains on the surface of the grinding disk is taken into account, and finally the *MRR* model of DSPG is established. This model predicts the *MRR* of a sapphire substrate in a DSPG process, providing a theoretical basis for the quantitative assessment of the *MRR* required in industrial applications.

## 2. Modeling of the Material Removal Rate

**Figure 1:**
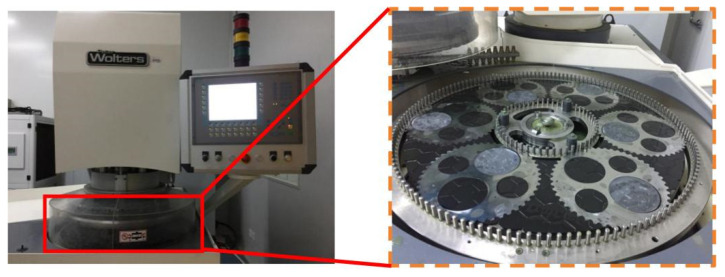
Peter Wolters AC700 double-sided grinding machine.

DSPG involves the use of the Peter Wolters AC700 double-sided grinding machine (Manufactured by Peter Wolters, Germany), employing solid diamond grinding wheels to meticulously grind sapphire substrates. This intricate process, depicted in Figure 1, achieves material removal from the substrate surface through the dynamic interplay between grinding particles and the substrate, propelled by the grinding pressure exerted by the upper grinding plate. The volume of material removed hinges directly on the grinding trajectory’s length as abrasive particles traverse the substrate surface. Given that these abrasive particles are firmly anchored to the grinding plate, the trajectory each particle follows is predetermined once processing parameters are set. Consequently, by examining the length of these grinding trajectories and understanding the theoretical indentation depth of individual abrasive particles, one can ascertain the volume of material removed during the grinding process. Therefore, during the model-building process, it is first necessary to determine the effective number of abrasive grains, as well as to obtain the theoretical indentation depth of individual grains. Through kinematic analysis, the complete trajectory length of individual particles is calculated. These calculations provide crucial insights into the material removal process, allowing for a deeper understanding of the mechanisms involved. It should be noted that the subsequent simulations in this paper refer to numerical solutions based on the model of the *MRR* and performed using the Python program (Python version: 3.10.11).

### 2.1. Effective Number of Abrasive Particles per Unit Area

During the processing phase, when an abrasive particle reaches a critical emergence height, it engages with the workpiece surface, initiating material removal through a scratching mechanism. This interaction with the effective abrasive particle is pivotal in the machining process. To accurately calculate the number of effective abrasive grains contained in a unit area of the surface of an abrasive disk, the height of exposure of these particles is an important metric. By evaluating the emergence height, one can discern the proportion of effective versus ineffective abrasive particles, thus optimizing the grinding efficiency and the *MRR* [29,30,31].

**Figure 2:**
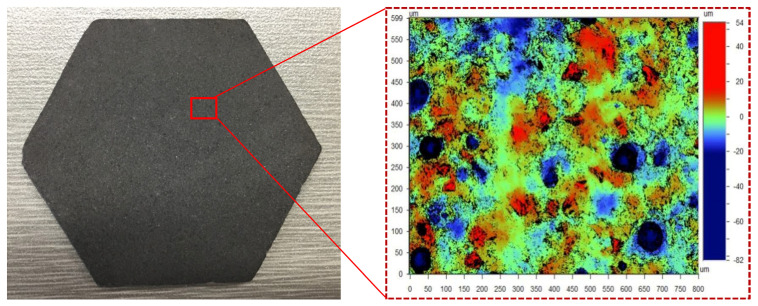
Distribution of abrasive particles on the grinding block surface.

**Figure 3:**
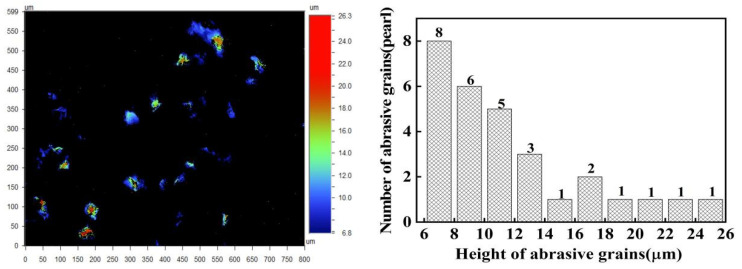
Distribution of the exposed heights of abrasive particles on the grinding plate surface.

Figure 2, obtained using the Wyko NT9300 white light interferometer, shows the abrasive particle distribution on the grinding wheel surface. The particles appear as a red, lumpy pattern, randomly interspersed within the bonding material, highlighting their uneven exposure on the wheel’s surface. Notably, besides these protruding abrasive particles and the bonding material above the reference plane, the surface also features significant hole structures of considerable depth and width. To accurately determine the effective abrasive particles, Wyko NT9300 software was employed to isolate the exposed height of these particles, as depicted in Figure 2. Through this process, the bonding material and hole structures were filtered out. The resultant data, shown in Figure 3, provide a clear representation of the effective abrasive particles, enabling a more precise analysis of their distribution and functionality in the grinding process.

The total count of exposed abrasive particles on the grinding wheel amounted to 29, with their exposed heights ranging from 6.8 μm to 26.3 μm. Notably, as the exposed height increased, the number of abrasive particles exhibited a declining trend. The largest cluster of particles, totaling eight, had exposure heights between 6 μm and 8 μm. Beyond 14 μm, the count of abrasive particles diminished sharply, with only one to two particles appearing for every 2 μm increment within the 14 μm to 26 μm range. The total number of particles within this exposure height bracket was a mere seven. Furthermore, no abrasive particles had an exposure height exceeding 26 μm, highlighting the pronounced scarcity of highly protruding particles.

**Figure 4:**
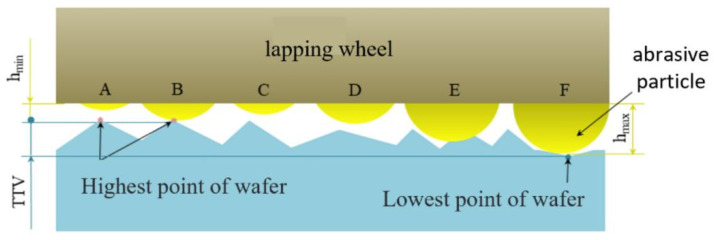
Contact model between the abrasive and the wafer.

Due to variations in the emergence height of abrasive particles and the thickness deviation (TTV) of the substrate surface, some particles with lower emergence heights fail to make contact with the substrate surface. By disregarding the effects of substrate curvature and warpage and assuming that each abrasive particle is spherical, the contact model between an abrasive particle and the substrate is depicted in Figure 4. In the limit state, when the abrasive particle with the maximum exposure height (*h_max_*) is in contact with the substrate’s lowest point, the particle with the minimum exposure height (*h_min_*) touches the substrate’s highest point. Particles with exposure heights below *h_min_* are unable to contact the substrate surface, thus failing to contribute to material removal. This contact dynamic highlights the critical role of particle emergence height in the effectiveness of the grinding process.

According to the assumptions of the contact model in Figure 4, the effective quantity of abrasives per unit area of the grinding plate, N_ap_, can be determined. The maximum exposure height of abrasives on the surface of lapping wheel is approximately 26 μm, while the thickness deviation (TTV) of the substrate surface is about 8 μm. For abrasive particles to effectively engage with the substrate surface, they must have an exposed height of at least 18 μm. By applying this condition to the software provided with the Wyko NT9300, we determined that the effective abrasive particle number, N_ap_, per unit area of the grinding wheel surface is 5.26 particles/mm^2^. To ensure accuracy, the average quantity of effective abrasives across ten points on the lapping wheel was calculated, yielding the effective abrasive particle number per unit area of the grinding plate. This refined measurement underscores the importance of precise particle height in optimizing the grinding process.

### 2.2. Depth of Indentation of a Single Abrasive Particle

#### 2.2.1. Effective Pressure-Bearing Area of the Substrate

**Figure 5:**
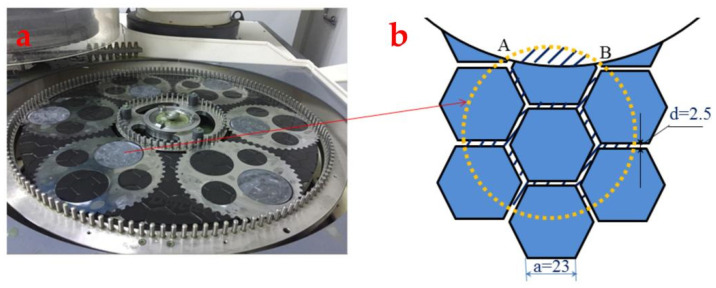
The contact area between the workpiece and the lapping grinding wheel: (**a**) the position of the workpiece on the grinding wheel and (**b**) a schematic diagram of the contact between the workpiece and the millstone.

Since the grinding plate is a patchwork of small square hexagonal grinding blocks, the substrate piece only comes into contact with abrasives in the effective area. This research focuses on double-sided planetary grinding, particularly involving a 4-inch sapphire substrate (radius R ≈ 100 mm) that had undergone line cutting. Illustrated in Figure 5a is the contact between this substrate and the grinding wheel. On the grinding plate, six sapphire substrates were arranged, ensuring even distribution. Figure 5b provides a schematic detailing the contact of an individual substrate with the grinding plate.

The effective pressure-bearing area *S_a_* of a single sapphire substrate is:

(1) $${S_{a}=S}_{w}-S_{1}-S_{2}=\pi R^{2}-d\cdot a\cdot n-{2[R}^{2}\cdot \arcsin\left(\frac{d_{\mathrm{AB}}}{2R}\right)-\frac{1}{2}\cdot d_{\mathrm{AB}}\cdot \sqrt{R^{2}-({\frac{d_{\mathrm{AB}}}{2})}^{2}})]$$

where S_w_ refers to the area of a single sapphire substrate, S_1_ refers to the joint area of the grinding block at the coverage of a single sapphire substrate, and S_2_ refers to the area where the substrate is placed beyond the grinding plate area.

#### 2.2.2. Theoretical Pressure on a Single Abrasive Particle

Grinding pressure is applied by pressurizing the upper grinding plate, and the effective abrasive particles contacting the effective pressure-bearing area of the substrate bear the applied load. Therefore, the theoretical pressure *P_s_* borne by a single abrasive particle is:

(2) $$P_{s}=\frac{F}{m\cdot S_{a}\cdot N_{\mathrm{ap}}}$$

where *F*—grinding pressure; m—number of substrates; *S_a_*—effective pressure-bearing area of a single substrate; and *N_ap_*—effective number of abrasive particles per unit area. The grinding pressure was set to 1000 N. The theoretical pressure of a single abrasive particle on the grinding wheel was 4.616 mN.

#### 2.2.3. Depth of Indentation of a Single Abrasive Particle

The indentation depth *d_H_* of a single abrasive particle pressed into a sapphire substrate under different loads is different, and both the indentation depth of a single crystal, C-faced sapphire under different loads and the scale effect exhibited by the material during the change in the indentation depth were thoroughly investigated by the Key Laboratory of Special Environmental Composites Technology of Harbin Institute of Technology in 2013 using a Boeing indenter [32].

Assuming that all effective grinding particles on the plate maintain a uniform height, it follows that each particle bears an identical theoretical load. Consequently, the indentation depth of each particle into the sapphire substrate remains consistent. From the data presented in [17,28] and the calculation of the theoretical pressure of a single particle, it can be concluded that, when the grinding pressure is 1000 N, the indentation depth of the particle on the grinding wheel into the sapphire substrate is about 50 nm, which increases when the grinding pressure increases.

### 2.3. Total Length of the Trajectory of the Abrasive Particles

#### 2.3.1. Trajectory Length of a Single Abrasive Particle

The equation of the trajectory left by a single abrasive was given by our previous research results as follows [1]:

(3) $$\left\{\begin{array}{c} X_{P\prime }=R_{P}\cos\left\{\left[\omega_{2}-\frac{2Z_{1}Z_{2}-Z_{1}Z_{3}}{Z_{2}(Z_{1}+Z_{3})}\omega_{1}\right]t+\varphi \right\}-L\cos\left[\frac{Z_{1}(Z_{2}-Z_{3})}{Z_{2}(Z_{1}+Z_{3})}\omega_{1}t\right]\\ Y_{P\prime }=R_{P}\sin\left\{\left[\omega_{2}-\frac{2Z_{1}Z_{2}-Z_{1}Z_{3}}{Z_{2}(Z_{1}+Z_{3})}\omega_{1}\right]t+\varphi \right\}+L\sin\left[\frac{Z_{1}(Z_{2}-Z_{3})}{Z_{2}(Z_{1}+Z_{3})}\omega_{1}t\right] \end{array}\right.$$

where *R_P_*—the distance between the abrasive particles and the sun gear center; ∅—the initial phase angle of abrasive particles; *L*—the center distance between the sun gear and the planet gear clamp; $\omega_{1}$—the sun gear angular velocity; $\omega_{2}$—the angular speed of the grinding wheel; $\omega_{3}$—the angular speed of the planetary gear clamp; Z_1_—the tooth number of sun gear; Z_2_—the tooth number of planetary gear clamps; and Z_3_—the tooth number of the fixed gear ring.

The derivatives of the above equation, *X*_*P*′_ and *Y*_*P*′_, can be used to obtain the partial velocities *v_x_* and *v_y_* of a single abrasive particle in the *x* and *y* directions, respectively. The instantaneous velocity *v* of the abrasive particle is:

(4) $$v=\sqrt{{v_{x}}^{2}+{v_{y}}^{2}}=\sqrt{{R_{P}}^{2}\cdot {A_{1}}^{2}+L^{2}\cdot {A_{3}}^{2}-2R_{P}\cdot A_{2}\cdot A_{3}\cdot L\cdot \cos\left(A_{2}-A_{4}\right)}$$

where

$${\;A}_{1}=\omega_{2}-\frac{Z_{1}\cdot Z_{3}\cdot \omega_{1}}{Z_{2}\left(Z_{1}+Z_{3}\right)};$$

$${\;A}_{2}=\omega_{2}t-\frac{Z_{1}\cdot Z_{3}\cdot \omega_{1}}{Z_{2}\left(Z_{1}+Z_{3}\right)}+\varphi ;\;$$

$${\;A}_{3\;}=\frac{Z_{1}\cdot (Z_{2}-Z_{3})\cdot Z_{3}\cdot \omega_{1}}{Z_{2}\cdot (Z_{1}+Z_{3})};$$

$${\;A}_{4}=\frac{Z_{1}\cdot (Z_{2}-Z_{3})\cdot \omega_{1}\cdot t}{Z_{2}\cdot (Z_{1}+Z_{3})};$$

Then, the trajectory length of a single abrasive particle *L_i_* is:

(5) $$L_{i}=\int_{0}^{t}vdt=\int_{0}^{t}\sqrt{{R_{P}}^{2}\cdot {A_{1}}^{2}+L^{2}\cdot {A_{3}}^{2}-2R_{P}\cdot A_{2}\cdot A_{3}\cdot L\cdot \cos\left(A_{2}-A_{4}\right)}dt$$

#### 2.3.2. Total Length of the Trajectory of Multiple Abrasive Particles on the Substrate

In the actual machining process, all of the effective abrasive particles on the grinding wheel surface participate in the grinding process. To determine the theoretical material removal rate, it is essential to calculate the total trajectory length *L_T_* traced by these effective abrasive particles on the substrate surface, as detailed below:

(6) $$L_{T}=\sum_{1}^{N_{T}}L_{i}$$

**Figure 6:**
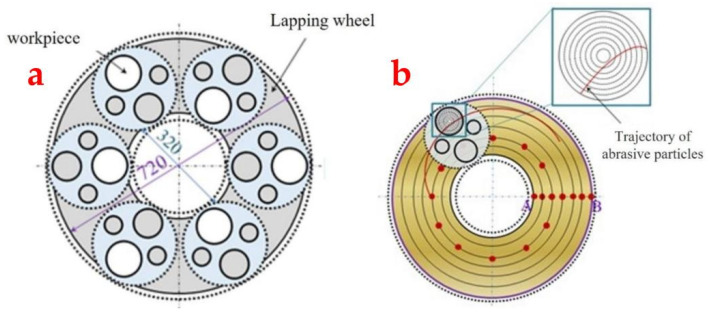
(**a**) Diagram of the substrate distribution on the grinding wheel. (**b**) The abrasive sampling point and the division of the substrate in concentric circles.

The trajectories left by abrasive particles at various positions on the grinding wheel surface exhibit significant variation. However, particles at concentric positions on the grinding wheel produce similar track shapes on the substrate. To comprehensively assess the impact of abrasive particle positions on trajectory length, we analyzed a series of concentric circles, spaced 1 mm apart radially on the grinding wheel. Additionally, we considered one abrasive point every 1 mm along these circles to capture detailed trajectory information for each abrasive point on the substrate surface. During actual processing, the substrate rotates within the mold. To accurately calculate trajectory length and account for the influence of substrate rotation, we divided the substrate into ten circular regions with equal radial distances. Figure 6 illustrates the abrasive sampling points and substrate divisions on the grinding plate.

As depicted in Figure 6b, abrasive particles carve out trajectory curves of varying lengths across different concentric circles on the substrate surface. To compute these, one can utilize the coordinate data of abrasive trajectory points to pinpoint the circular region where the abrasive particle resides at any given moment. By setting a sufficiently small time step, the distance between successive points can be approximated as the length of a straight line segment. Consequently, the total trajectory length is obtained by summing the lengths of trajectories across all regions.

**Figure 7:**
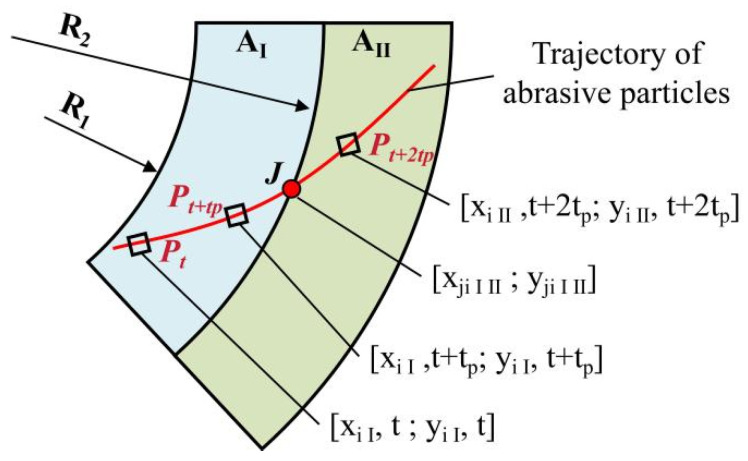
The distribution of the trace points in the partition of the substrate surface.

Figure 7 illustrates the trajectory distribution of abrasive grain points within the surface partition of the substrate. Let the time step be *t_p_*, and the position of the abrasive particle *P* on the substrate at moment *t* be the *P_t_* point in the A_I_ annular region. After one time step *t_p_*, the *P* point is still in the *P_t*+*tp_* point in the A_I_ annular region, but after 2*t_p_*, the *P* point enters the *P_t*+2*tp_* point in the A_II_ annular region. From this observation, it becomes evident that the instantaneous velocity changes of abrasive particles result in varying trajectory lengths for the same time step. Consequently, two scenarios arise: either two adjacent trajectory points lie within the same annular region, or they occupy different annular regions.

If two neighboring points fall within the same circular region (such as A_I_) on the surface of the grinding wheel, the length of the trajectory between the two points is calculated within the same region. The length of this trajectory Δ*S_iI*,*t*+*tp_* can be estimated by the following equation.

(7) $${∆S}_{iI,t+\mathrm{tp}}=\sqrt{{\left(x_{iI,t+t_{p}}-x_{iI,t}\right)}^{2}+{\left(y_{iI,t+t_{p}}-y_{iI,t}\right)}^{2}}$$

If two adjacent points fall within different circular regions, such as points *P_t*+*tp_* and *P_t*+2*tp_*, as shown in Figure 7, at this time, consider the dividing point *J* of the two circular regions as the separating point and count the lengths of the two trajectories into their corresponding circular regions, i.e., the trajectory lengths between the point starting from *Pt*+*tp* and reaching the intersection point *J*, between the trajectory and the region boundary, are counted in region A_I_, and the trajectory lengths of the remaining part are counted in region A_II_, where the trajectory length Δ*S_iI*,*t_* in region A_I_ is:

(8) $${∆S}_{iI,\;t}=\sqrt{{\left(x_{iI,t+t_{p}}-x_{\mathrm{Ji}\mathrm{III}}\right)}^{2}+{\left(y_{iI,t+t_{p}}-y_{\mathrm{Ji}\mathrm{III}}\right)}^{2}}$$

The trajectory length Δ*S_iII*,*t_* in area A_II_ is:

(9) $${∆S}_{i\mathrm{II},\;t}=\sqrt{{\left(x_{i\mathrm{II},t+2t_{p}}-x_{\mathrm{Ji}\mathrm{III}}\right)}^{2}+{\left(y_{i\mathrm{II},t+2t_{p}}-y_{\mathrm{Ji}\mathrm{III}}\right)}^{2}}$$

Consider *T* as the total processing time; then, in *T* time, the trajectory length *S_iI_* of a point *P* on the grinding wheel in the ring area of substrate A_I_ is:

(10) $$S_{iI}=\sum_{t=0}^{T}{∆S}_{iI,\;t}$$

We overlaid the trajectory length of all points (*P*_1_, *P*_2_, …, *P*_N_) on the workpiece in the annular area A_I_ to obtain the total trajectory length of the abrasive particles in this area:

(11) $$S_{I}=\sum_{i=0}^{N}S_{iI}$$

The total trajectory length *L_T_* of the substrate surface can be obtained by summing the trajectory lengths in the ten annular regions on the substrate:

(12) $$L_{T}=\sum_{i=I}^{X}S_{i}$$

### 2.4. Groove Repetition Rate

**Figure 8:**
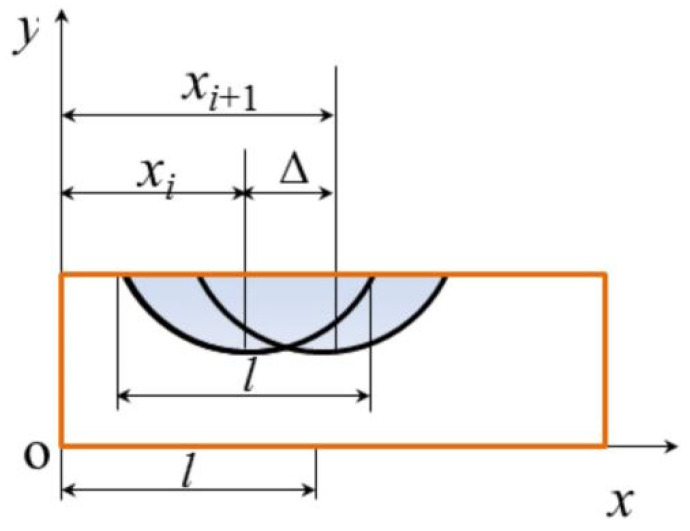
Spacing of adjacent grooves on the substrate surface.

During the process of DSPG, the grooves scratched by abrasives on the substrate surface overlap each other. To obtain a material removal rate that closely reflects the real grinding scenario, it is essential to account for and eliminate the volume repetition caused by these overlapping grooves. Calculating trench repetition requires determining the spacing between two adjacent trenches. Figure 8 illustrates the overlap of two adjacent grooves on the substrate surface. Assume the spacing between trenches is Δ, with the substrate’s center point as the coordinate origin for a two-dimensional coordinate system. Let the *x* coordinates of the center points of the two grooves be *x_i_* and *x_i*+*_*_1_.

$$\mathrm{Let}\left\{\begin{array}{c} l_{1}=x_{i+1}-x_{i}\\ l_{2}=x_{i} \end{array}\right.$$

The probability density function of *l*_1_ is:

(13) $$f_{l_{1}}\left(l_{1}\right)=\int_{-\infty }^{+\infty }f_{x_{i+1}}\left(x_{i+1}\right)f_{x_{i}}\left(x_{i+1}-l_{1}\right)dx_{i+1\;}$$

Furthermore, $f_{l_{1}}\left(l_{1}\right)$ can also be written as

(14) $$f_{l_{1}}\left(l_{1}\right)=\left\{\begin{array}{c} \frac{1}{l^{2}}\int_{l_{1}}^{l}dx_{i+1}=\frac{l\;-\;l_{1}}{l^{2}},\;0\leq l_{1}\leq l\\ \frac{1}{l^{2}}\int_{0}^{l+l_{1}}dx_{i+1}=\frac{l+l_{1}}{l^{2}},\;-l_{1}\leq l_{1}\leq 0 \end{array}\right.$$

Therefore, the mathematical expectation of the Δ is:

(15) $$E\left(∆\right)=\int_{0}^{l}∆f_{∆}\left(∆\right)d∆=\frac{l}{3}$$

**Figure 9:**
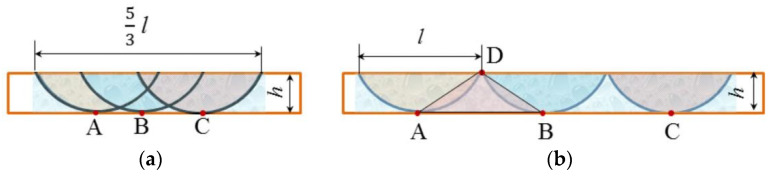
The difference in cross-sectional area between the repeated and unrepeated grooves. (**a**) The cross-sectional area when the groove is repeated, *S*′, and (**b**) the cross-sectional area when the grooves are not repeated, *S*.

Taking the three grooves on the substrate as an example, Figure 9 shows the difference in the sectional area of the three grooves when they are repeated and not repeated. Let the groove depth be *h*; from the above calculation, the total width of the three grooves is 5*l*/3 when the groove is repeated. Since *h* is in the nanometer level, the groove area can be approximately regarded as a rectangle. From this, it can be concluded that:

(16) $$\left\{\begin{array}{c} S^{\prime }\approx \frac{5}{3}lh\\ S\approx 3lh-\frac{1}{2}lh \end{array}\right.$$

Therefore, the trench repetition rate η is approximately:

(17) $$\eta =1-\frac{S^{\prime }}{S}\;\approx \;\frac{1}{3}$$

### 2.5. Material Removal Rate Model

**Figure 10:**
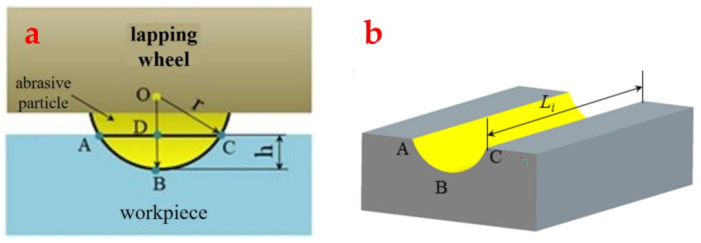
Theoretical model of the abrasive pressing into the surface of the workpiece. (**a**) a single abrasive is pressed into the surface of the workpiece and (**b**) the groove formed on the surface of the workpiece after abrasive scratches.

Since the abrasive particles are fixed to the grinding wheel’s surface, the grinding trajectory of each particle scraping across the substrate is fixed once the processing parameters are set. Consequently, the volume of material removed during scratching can be theoretically determined by analyzing the trajectory length of the abrasives, provided the indentation depth of an individual particle is known. Figure 10 illustrates the formation of a groove after a single abrasive grain is scratched on the surface of a workpiece to remove material. To determine the material removal volume based on trajectory length, we propose three hypotheses.

**Assumption** **1.** *Since grinding processes produce brittle chips on the material surface, it is assumed that the abrasive particles are spherical. This assumption aims to ensure the calculated value approximates the volume of the groove left by the abrasive particles on the substrate after material removal.*

**Assumption** **2.** *The abrasives on the grinding wheel are uniform in size and evenly distributed.*

**Assumption** **3.** *The substrate surface is removed by micro-cutting, and each abrasive particle has the same groove shape on the workpiece surface, and the groove overlap rate is set to η.*

(18) $$V_{i}=S_{\mathrm{ABC}}\cdot L_{i}=[R^{2}\cdot \arccos\left(\frac{R-d_{H}}{R}\right)-\left(R-d_{H}\right)\sqrt{2R\cdot d_{H}{-R}^{2}}]\cdot L_{i}$$

The total volume of the material removed from the substrate by *N_T_* effective abrasive particles is

(19) $$V_{T}=(1-\eta ){\cdot S}_{\mathrm{ABC}}\cdot L_{i}\cdot \sum_{1}^{N_{T}}L_{i}$$

From this, it can be seen that, if the grinding time is t, the *MRR* of one side of the substrate is:

(20) $$\mathrm{MRR}=\frac{V_{T}}{S_{w}\cdot t}=\frac{(1-\eta ){\cdot S}_{\mathrm{ABC}}\cdot \sum_{1}^{N_{T}}L_{i}}{S_{w}\cdot t}$$

## 3. Experimental Verification

### 3.1. Design of the Grinding Experiments

The experimental machine tool used was an AC 700-F CNC high-precision double-disk machine (Manufactured by Peter Wolters, Germany). The substrate used in the experiments was a 4-inch C-faced sapphire substrate (size: Φ101.6 mm × 780 μm) after wire-cutting, and the initial surface quality was required to have a high consistency. The substrate was weighed before and after processing, and the *MRR* was calculated based on the weight difference and processing time.

### 3.2. Comparison of the Simulation and Experimental Results

**Figure 11:**
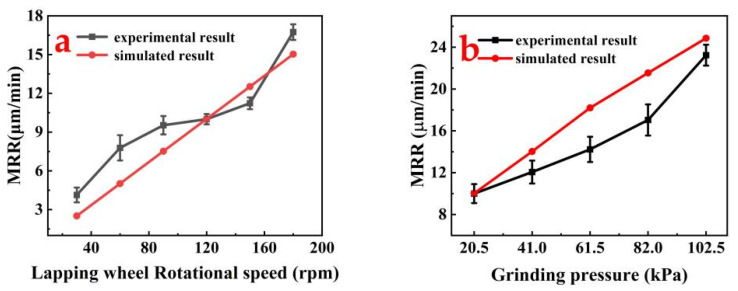
Influence of grinding parameters on the *MRR*: (**a**) influence of grinding wheel speed on the *MRR* and (**b**) influence of grinding pressure on the *MRR*.

The *MRR* of the substrate under different processing parameters was obtained by incorporating the repetition rate of groove *η* and total trajectory length *L_T_* into Equation (20). To verify the reliability of the simulated *MRR* values, the simulated *MRR* values were compared with the experimental *MRR* values under two machining conditions, namely, grinding speed and grinding pressure, and the comparison results are shown in Figure 11.

Grinding speed has an effect on the *MRR* and to further investigate the relationship between them, the value of grinding pressure was fixed at 20.5 kPa and the grinding speed was 30~180 rpm, with the speed gradient set at 30 rpm for a total of six speed values. The *MRR* values at the six grinding speeds were obtained using Equation (20) and then compared with the experimental *MRR* values under these six grinding conditions. Figure 11a illustrates the results of the comparison.

The *MRR* simulation results revealed a linear increase with the rising speed of the grinding plate, ranging from 2 to 16 μm/min. Similarly, the experimental *MRR* values exhibited an approximately linear upward trend, spanning from 4 to 18 μm/min. The error margin between the simulation and experimental results varied between 0.2% and 39.2%, with the maximum discrepancies occurring at speeds of 30 rpm and 60 rpm. Despite some deviations due to random factors in the experimental process, the close alignment of the simulation and experimental trends indicates high reliability in the simulation results. This consistency underscores the robustness of the simulations, notwithstanding the minor deviations observed.

The grinding pressure also has an effect on the *MRR*; therefore, five different grinding pressures were set as 20.5 KPa, 41 kPa, 61.5 kPa, 82 kPa, and 102.5 kPa, and the speed of the grinding plate was kept constant at 120 rpm. The *MRR* values under the five grinding pressures were obtained using Equation (20) and compared with the experimental *MRR* values under these five grinding pressures. Figure 11b illustrates the results of the comparison.

The simulated *MRR* results exhibited a linear increase with rising grinding pressure, with values ranging from 9 μm/min to 25 μm/min. Similarly, the experimental *MRR* values showed an approximately linear upward trend with increasing grinding wheel speed. The error margin between the simulation and experimental results spanned from 0.2% to 21.8%, with values also distributed between 9 μm/min and 25 μm/min. Comparing these values and trends reveals a clear consistency between the simulated and experimental *MRR* results, underscoring the reliability of the simulation. The simulated results have high reliability. However, with the increase in the grinding pressure during the experiment, the effective number of abrasive particles changes randomly due to the random distribution of the emergence height of abrasives, causing a deviation in the abrasive depth of the cut from its ideal setting, and finally leads to a large difference between the simulated and experimental values of the *MRR* at the grinding pressures of 61.5 kPa and 82 kPa. From the results above, it is evident that the simulated and experimental *MRR* values under the varying conditions of grinding wheel speed and grinding pressure align closely. This high degree of agreement confirms the reliability of the simulation results.

### 3.3. Optimization of the Removal Rate of the Upper and Lower Surface Material

**Figure 12:**
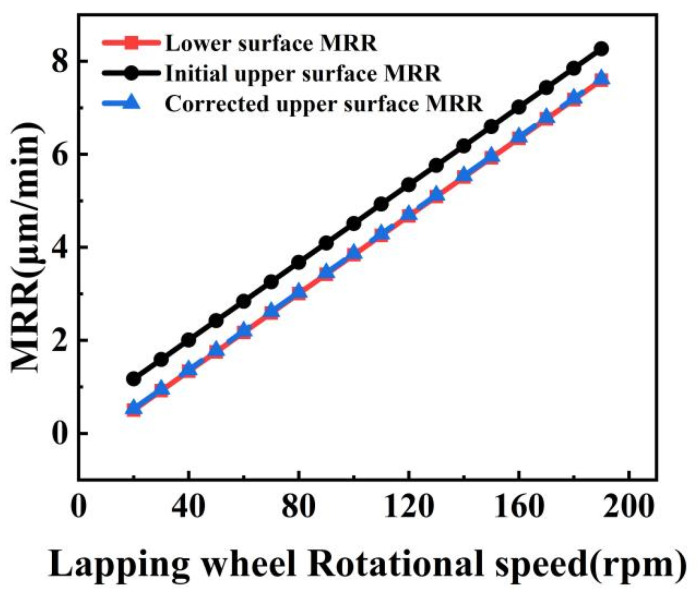
*MRR* difference between the two workpiece surfaces.

Both grinding plates remove material from two surfaces of the substrate simultaneously during the process of DSPG. As the plates rotate in opposite directions, even when their rotational speeds are identical, the abrasive particles trace different trajectory lengths on the two surfaces. This discrepancy leads to varying amounts of material removed from each surface. To investigate the *MRR* differences between the substrate’s upper and lower surfaces, simulations were conducted at various grinding speeds. The results of these simulations are presented in Figure 12.

Theoretically, the *MRR* of each surface of the substrate, as well as the total *MRR*, is linearly dependent on the grinding speed. It is worth noting that under the same machining conditions, the two surfaces of the sapphire substrate do not have the same *MRR*. This phenomenon occurs because the upper grinding plate rotates in the opposite direction to the substrate, while the lower grinding plate rotates in the same direction as the substrate. Consequently, there is a disparity in the velocity of the abrasive grains on the two surfaces of the substrate. The abrasive grains exhibit a higher velocity on the upper surface of the workpiece.

A significant disparity in the *MRR* between the upper and lower surfaces results in stress differences post machining. This imbalance enlarges the bow and warp values of the substrate, negatively impacting the accuracy of the substrate’s face shape. Therefore, it is crucial to minimize the stress difference by reducing the *MRR* disparity between the two surfaces of the substrate.

The *MRR* difference between the two surfaces of the substrate primarily arises from the significant relative speed between the top grinding plate and the substrate’s upper surface. To mitigate this disparity, it is essential to reduce the speed of the upper grinding plate while maintaining the lower grinding wheel’s speed. Let *n*_0_ be the corrective speed. Calculations indicate that when the upper grinding wheel speed is 16 rpm slower than the lower grinding wheel speed, the *MRR* of both surfaces becomes nearly equal, achieving balance at *n*_0_ = 16rpm. Denote the upper grinding wheel speed as *n*_1_ and the lower grinding wheel speed as *n*_2_. To minimize the relative speed difference between the top and bottom grinding plates and the workpiece, the following relationship must hold: *n*_1_ = *n*_2_ + *n*_0_. Given the lower wheel speed, the corresponding upper wheel speed can thus be determined.

To achieve a substrate surface with minimal *MRR* difference between two surfaces, it is necessary to adjust the speed of the upper grinding plate appropriately. Following the adjustments to the upper grinding plate speed as specified in Table 1, the *MRR* for both surfaces was recalculated. The results, depicted in Figure 12, demonstrate that after these adjustments, the *MRR* of two surfaces achieved parity. This alignment significantly enhances the accuracy of the wafer surface’s face shape.

## 4. Conclusions

On the basis of analyzing the distribution of abrasive particles, this article established a model of *MRR* through the contact of particles and the substrate based on the trajectory length of particles, through which the simulation of the *MRR* was achieved more accurately. The following four conclusions were obtained in this paper:

(1) The model of the *MRR* for DSPG was developed based on the trajectory length of abrasive particles and grinding experiments on sapphire substrates and verified the reliability of the model of *MRR*.
(2) The *MRR* of double-sided planetary grinding under different conditions can be predicted using this model, and the optimization of the process of DSPG can be carried out using this model.
(3) Based on the model of *MRR*, the process parameters can be optimized to achieve the same *MRR* on two surfaces of the workpiece during the process of DSPG and enhance the machining accuracy of the surface.
(4) The method of the *MRR* model based on the trajectory length of particles is also applicable to the double-sided planetary grinding of other materials.

The *MRR* model developed in this paper does not take into account the chemical reactions occurring on the sapphire surface that promote material removal, in addition to the thermal effect of the abrasive particles in contact with the sapphire surface, which also affects material removal. The model of *MRR* considering the complex grinding environment should be further investigated.

## Figures and Tables

**Table 1:** Rotational speed values of the top and bottom disks.

*n*_1_ (rpm)	20	30	40	50	60	70	80	90	100
*n*_2_ (rpm)	4	14	24	34	44	54	64	74	84
*n*_1_ (rpm)	110	120	130	140	150	160	170	180	190
*n*_2_ (rpm)	94	104	114	124	134	144	154	164	174

## Data Availability

The original contributions presented in the study are included in the article, further inquiries can be directed to the corresponding author.
